# Morphology of adult and juvenile instars of *Galumna obvia* (Acari, Oribatida, Galumnidae), with discussion of its taxonomic status

**DOI:** 10.3897/zookeys.357.6404

**Published:** 2013-11-29

**Authors:** Sergey G. Ermilov, Gerd Weigmann, Andrei V. Tolstikov

**Affiliations:** 1 Tyumen State University, Tyumen, Russia; 2 Free University of Berlin, Institute of Zoology, Berlin, Germany

**Keywords:** Oribatida, *Galumna obvia*, morphology, supplementary description, juvenile instars, ontogeny, insertions of lamellar setae

## Abstract

The adult instar of the oribatid mite, *Galumna obvia* (Berlese, 1914), is redescribed in detail, on the basis of specimens from Finland. The morphology of juvenile instars of *G. obvia* is described and illustrated for the first time, and compared to that of other species of the family Galumnidae. The position of the insertion of the lamellar seta in adults proved variable in studied European populations, being either on or medial to the lamellar line. Since the genera *Galumna* and *Pergalumna* are currently distinguished only by the relative positions of the seta and line, specimens of *G. obvia* in some populations show an intermediate situation between other studied *Galumna* species – with lamellar seta on or lateral of lamellar line – and *Pergalumna* with lamellar seta at a distinct distance medially of lamellar line. A detailed reevaluation of the two genera is needed.

## Introduction

The oribatid mite *Galumna obvia* (Oribatida, Galumnidae) was described by Berlese (1914) as *Oribata obvius*. This species has a semicosmopolitan distribution, being known from the Palearctic and Neotropical regions, i.e. from the USA, South Africa, Santa Elena Islands, Vietnam, and Hawaii (references were summarized by Subías 2004, updated 2013).

The original descriptionand several redescriptions of adult *Galumna obvia* (see below, *Taxonomic history*) were incomplete, lacking measurements of morphological structures and information about leg setation and solenidia, and morphology of the gnathosoma. Further, lateral and ventral views of the idiosoma, which have important traits in this family, were insufficiently studied and illustrated. The present paper provides a detailed description and illustrations of *Galumna obvia* on the basis of 10 specimens collected in Finland as a reference population. Our data and a literature review show variation in the insertion of lamellar setae, relative to the lamellar line; since this insertion is considered important in distinguishing *Galumna* from *Pergalumna*, we discuss the generic position of *Galumna obvia*.

Additionally, we described and illustrated the morphology of juvenile instars of *Galumna obvia*. The family Galumnidae comprises more than 450 species, however, the full series of juvenile instars has been studied in detail only for eight species: *Acrogalumna longiplumna* (Berlese, 1904) (Seniczak et al. 2012), *Allogalumna alamellae* (Jacot, 1935) (Seniczak et al. 2012), *Galumna alata* (Hermann, 1804) (Seniczak et al. 2012), *Galumna zachvatkini* Grishina, 1982 (Grishina 1982), *Pergalumna nervosa* (Berlese, 1914) (Sengbusch 1954; Seniczak 1972; Grishina 1977; Seniczak et al. 2012), *Pilogalumna crassiclava* (Berlese, 1914) (Seniczak and Seniczak 2007), *Pilogalumna ornatula* Grandjean, 1956 (Seniczak and Seniczak 2007), and *Pilogalumna tenuiclava* (Berlese, 1908) (Seniczak 1972; Seniczak and Seniczak 2007). In addition, Sengbusch (1954) briefly described all juvenile instars of *Galumna ithacensis* (Jacot, 1929).

Also, the juvenile instars have been described incompletely and/or illustrated in several species briefly, namely: *Acrogalumna longiplumna* (Grandjean 1935), *Dicatozetes numidicus* Bernini, 1984 (Bernini 1984), *Dicatozetes uropygium* (Grandjean, 1928) (Grandjean 1928), *Galumna alata* (Michael 1884), *Galumna louisianae* (Jacot, 1929) (Woodring 1965), *Galumna parva* Woodring, 1965 (Woodring 1965), *Galumna tarsipennata* Oudemans, 1914 (Travé 1970), *Galumna* sp. (Zachvatkin 1953), *Orthogalumna terebrantis* Wallwork, 1965 (Wallwork 1965), *Pergalumna nervosa* (Cooreman 1941), *Pergalumna emarginata* (Banks, 1895) (Rockett and Woodring 1966), *Pilogalumna ornatula* (Grandjean 1956a), *Pilogalumna tenuiclava* (Grandjean, 1933), and *Vaghia carinata* (Travé, 1955) (Travé 1955).

Grandjean (1953) summarized the main generic characteristics of juvenile instars of Galumnidae.

## Material and methods

Specimens of *Galumna obvia* were collected at the following locality: Finland, 64°24'10.78"N, 25°26'7.86"E, Päijänne National Park, Virmailansaari Island, near Padasjoki, 80 m a.s.l., Piceetum vaccinioso-hylocomiosum plant association, moss cover on stones and soil litter, 15.07.2013, collected by Andrei V. Tolstikov. The material collected in the field contained 10 adults, five larvae, two protonymphs and one deutonymph.

Comparative material for the taxonomic discussion originates from one Portuguese and some German locations:

- Ribeira de Aljezur, Atlantic coast area of West-Algarve, Portugal, 37.347°N, 8.846°W, floodplain forest, 2011. Weigmann’s collection (*Galumna tarsipennata*);
- River Oder Valley, Criewen; North-East Germany, 53.012°N, 14.233°E, moist deciduous forest, 1999. Weigmann’s collection (*Galumna obvia*);
- “Berlin 1”; Berlin-Lübars, 52.62°N, 13.37°E, moist meadow, 1986. Weigmann’s collection (*Galumna obvia*);
- “Berlin 2”; Postfenn, 52.498°N, 13.24°E, degraded moor, 1997. Weigmann’s collection (*Galumna obvia*);
- “Berlin 3”; Berlin-Spandau, Teufelsbruch, 52.579°N, 13.205°E, moor area, 1997. Weigmann’s collection (*Galumna obvia*, *Galumna alata*);
- “Berlin 4”; Berlin-Charlottenburg, 52.5°N, 13.35°E, park forests, 1995. Weigmann’s collection (*Galumna lanceata*);
- “Oldesloe”; Brenner Moor, near Oldesloe, Schleswig-Holstein, North-West Germany, 53.78°N, 10.33°E, salty moor complex, 1973. Weigmann’s collection (*Galumna obvia*).

Specimens were mounted in lactic acid on temporary cavity slides for measurement and illustration. All body measurements are presented in micrometers. Body length was measured in lateral view, from the tip of the rostrum to the posterior edge of the ventral plate. Notogastral width refers to the maximum width in dorsal aspect. Lengths of body setae were measured in lateral aspect. Formulae for leg setation are given in parentheses according to the sequence trochanter–femur–genu–tibia–tarsus (famulus included). Formulae for leg solenidia are given in square brackets according to the sequence genu–tibia–tarsus. General terminology used in this paper mostly follows that summarized by Grandjean (see Travé and Vachon for references), Weigmann (2006), and Norton and Behan-Pelletier (2009).

## Taxonomic history of *Galumna obvia* (Berlese, 1914)

*Oribates obvius* Berlese, 1914: 119, pl. 1: 1. (type locality: Florence, Italy)

*Galumna obvius*: Sellnick 1928 (9); Willmann 1931 (138, fig. 302).

*Galumna obvia*: Zachvatkin 1953 (158, fig. 65); Shaldybina 1975 (353, fig. 887); Mahunka 1992 (246); Mahunka and Mahunka-Papp 1995 (86, 210, fig. 161); Weigmann 2006 (373, fig. 197); Bayartogtokh 2011 (339, fig. 87E).

*Galumna obvium*: Pérez-Iñigo 1993 (77, fig. 25a).

*Galumna “elimata”* sensu van der Hammen 1952; nec C.L. Koch 1841: Aoki 1966 (774, figs 13–15); Sellnick 1960 (56); Wallwork 1977 (232, fig. 91c).

There is some confusion regarding the validity of *Galumna obvia*, which was declared as junior synonym of *Galumna elimata* (described as *Oribates elimatus* Koch, 1841 in CMA 31.5) firstly by Jacot (1929: 4) in the context of the discussion on the type species of *Zetes* Koch, 1835, a question which is not relevant for the synonymy of the species. The original figure of Koch’s *Oribates elimatus* shows clearly long interlamellar setae, but the original description of *Galumna obvia* by Berlese (1914, tav. 10: 1) shows clearly very short interlamellar setae, confirmed by the type study of Mahunka (1992) who refered to the redescription of Shaldybina (1975: 353, fig. 887) being in accordance to Berlese’s species. Berlese himself (Berlese 1914: 122, Tav. 10: 7) published his interpretation of *Galumna elimata* (Koch) with the remark that it is different from *Galumna obvia*. Mahunka (1992: 242, fig. 57) figured *Galumna elimata* Koch (sensu Berlese) after a slide in the Berlese collection and declared the specimen in Berlese’s slide 153/29 as lectotype. We follow the interpretations of Mahunka (1992) as did Weigmann (2006) that *Galumna elimata* Koch and *Galumna obvia* Berlese are distinct species. Consequently, the synonymization of *Galumna obvia* with *Galumna elimata* as senior synonym by Jacot (1929) must be rejected. The interpretations of van der Hammen (1952) and others (see above) under the name “*Galumna elimata*” are based on Jacot (1929) and refer to *Galumna obvia*.

### Supplementary description of adult *Galumna obvia*

Figs 1–20

**Figures 1–8.**
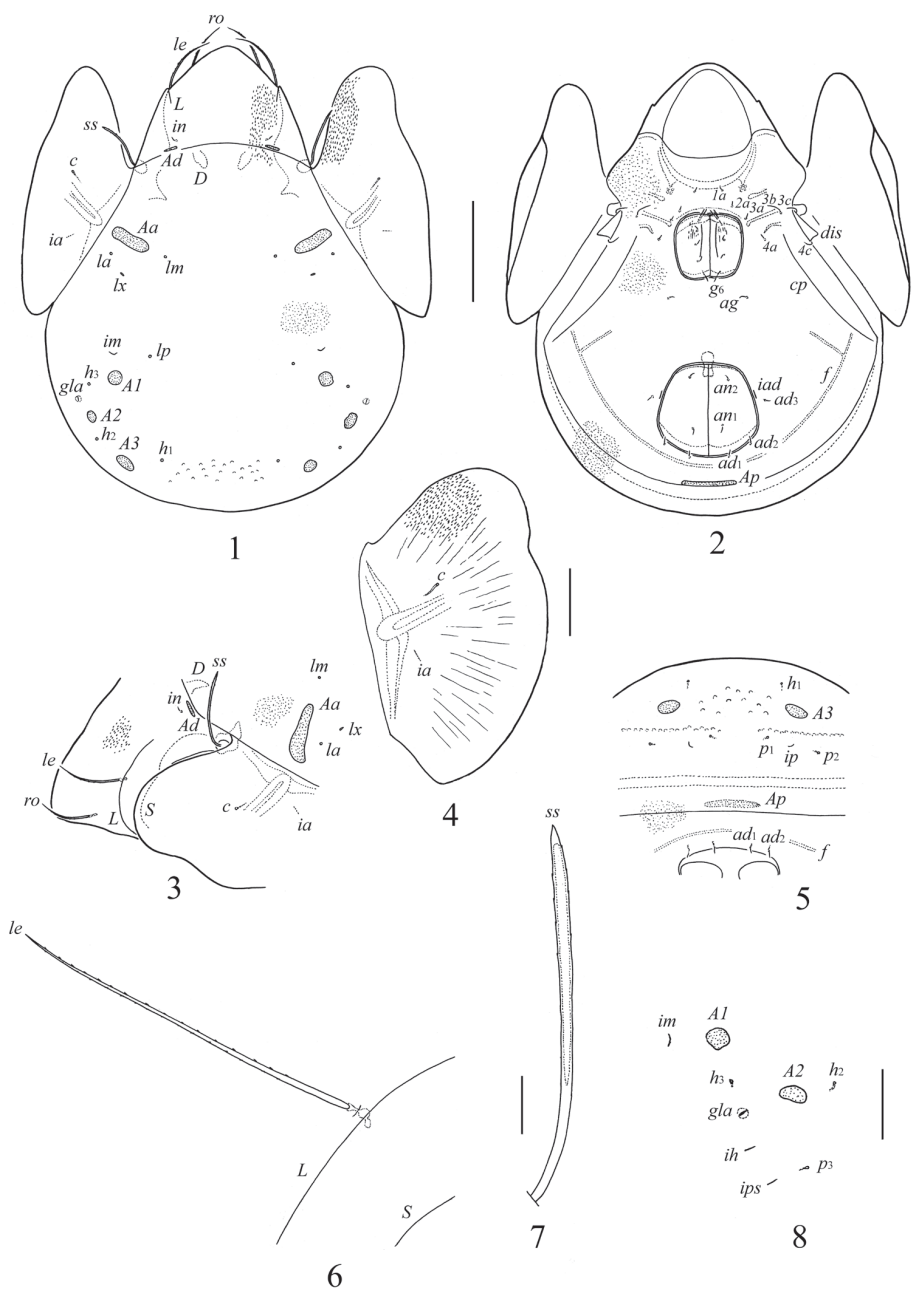
*Galumna obvia*, adult: **1** dorsal view **2** ventral view (gnathosoma and legs not shown) **3** anterior part of body, lateral view **4** pteromorph **5** posterior view **6** lamellar seta and parts of lamellar and sublamellar lines **7** sensillus **8** dorso-lateral part of notogaster, lateral view. Scale bars 200 μm (**1–3, 5**), 100 μm (**4**), 20 μm (**6–8**).

**Figures 9–20.**
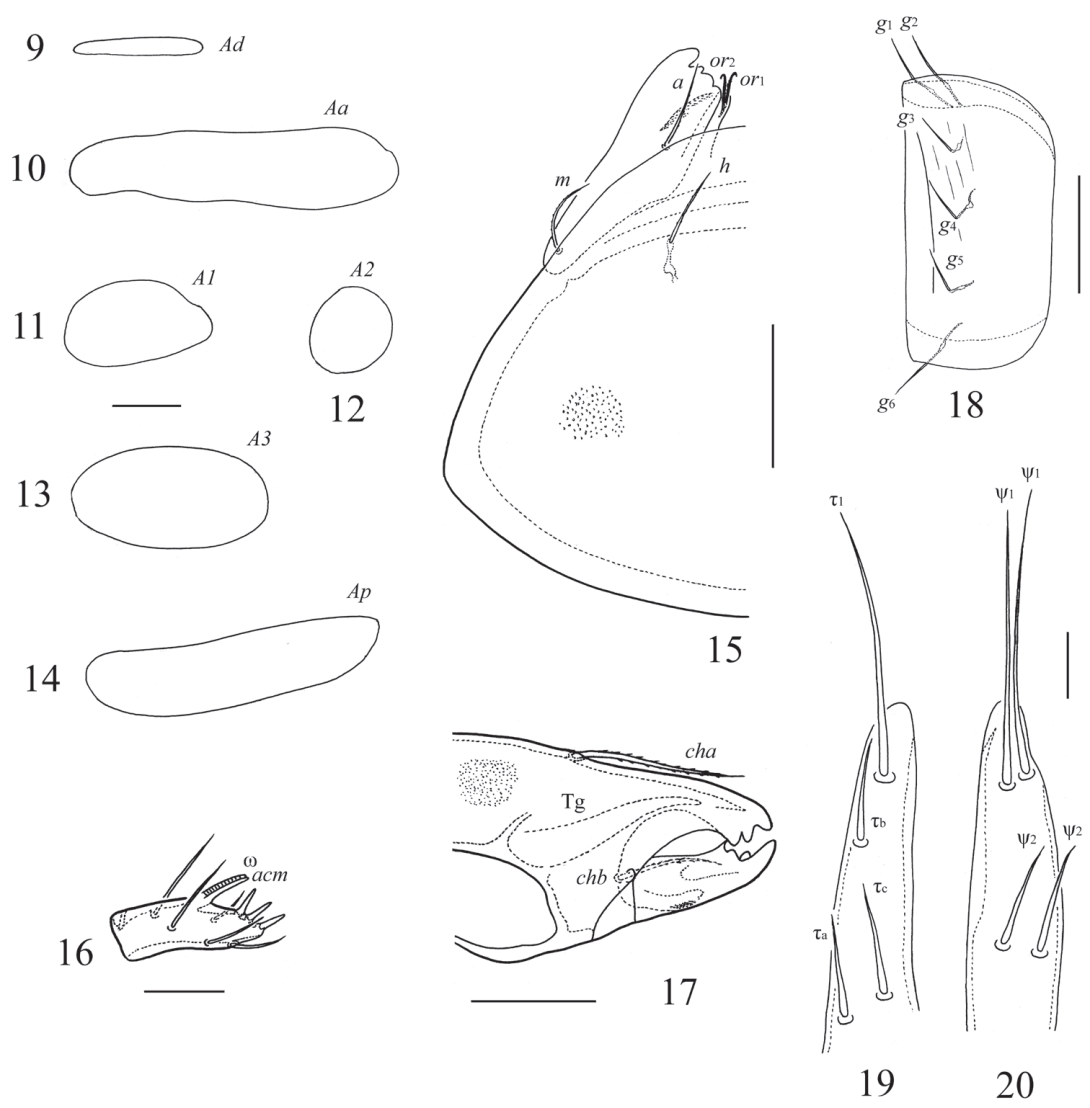
*Galumna obvia*, adult: **9** porose area *Ad* **10** porose area *Aa* **11** porose area *A1* **12** porose area *A2* **13** porose area *A3* **14** porose area *Ap* **15** subcapitulum, right half, ventral view **16** palptarsus **17** anterior part of chelicera **18** genital plate, left **19–20** lobes of ovipositor. Scale bars 20 μm (**9–14, 16, 19, 20**), 50 μm (**15, 17, 18**).

*Measurements*. Body length 846–898, width 630–647 (10 specimens, three males and seven females).

*Integument*. Body color brown to brownish-black. Body surface microfoveolate (well visible under high magnification); foveolae rounded (diameter up to 1) or represented by short lines (on prodorsum and pteromorphs). Posterior part of ventral plate with long, light furrow (*f*), located posterior and lateral to anal plates. Genital plates with one strong longitudinal fold located medial to genital setae; additional, short, weakly visible folds present in several specimens.

*Prodorsum*. Rostrum broadly rounded. Rostral (*ro*) and lamellar (*le*) setae setiform, barbed. Interlamellar setae (*in*) short, thin, smooth. Sensilli (*ss*) long, with weakly developed, elongate, barbed head pointed distally. Exobothridial setae absent. Relative length of prodorsal setae: *ss* ≈ *le* > *ro* > *in*; measurements given in Table 1. Lamellar (*L*) and sublamellar (*S*) lines distinct, parallel. Insertions of lamellar setae located medially to the lamellar lines, very close to them. Porose areas *Ad* (28–45 × 4–8), transversely oriented, thin, located posterolateral to interlamellar setae.

**Table 1.** Comparison of body setae measurements of *Galumna obvia* during ontogeny

Character	Larva	Protonymph	Deutonymph	Adult
	n=5	n=2	n=1	n=10
Length of prodorsal setae:				
– rostral setae	53–61	57–61	69	86–98
– lamellar setae	45–53	53–57	61	143–164
– interlamellar setae	32–36	36–45	41	8–12
– sensilli	73–82	77–86	102	143–164
– exobothridial setae	32–36	45–49	45	Absent
Length of gastronotic setae:				
– *c*_3_	45	45	45	Absent
– *h*_2_	20–24	4–6	8	Absent
– *p*_2_, *p*_3_	Absent	14–16	20	Absent
– other gastronotic setae	4	4–6	8	Absent
Length of epimeral setae	8–12	12–16	16	*3b*, *3c*, *4c* (28–36); *1a*, *2a*, *4a*, *4b* (12–20)
Length of anogenital setae:				
– genital setae	Absent	12–16	16	12–20
– aggenital setae	Absent	Absent	16	8–16
–anal setae	Absent	Absent	Absent	8–16
– adanal setae	Absent	Absent	16	8–16

*Notogaster*. Anterior notogastral margin well developed. Dorsophragmata (*D*) of medium size. Notogastral setae represented by 10 pairs of alveoli. Four pairs of porose areas present: *Aa* (77–131 × 16–32) transversly oriented, elliptical to weakly boot-shaped; *A1* (24–57 × 24–32) and *A2* (24–53 × 20–28) round or oval; *A3* (28–69 × 20–36) oval. All porose areas well visible, but without distinct margins. Alveoli of setae *la* inserted posterior to *Aa*. Median pore absent. All lyrifissures distinct; *im* located anterior to *A1*. Opisthonotal gland openings (*gla*) located anterolateral to *A2*.

*Gnathosoma*. Subcapitulum longer than wide (188–200 × 172–176). Subcapitular setae (*a*, *m*, *h*) similar in length (28–36), setiform, slightly barbed. Adoral setae (*or*_1_, *or*_2_) (16–20) setiform, barbed. Palps (147–155) with setation 0–2–1–3–9(+ω); solenidion straight. Chelicerae (225–241) with two setiform, barbed setae; *cha* (65–73) longer than *chb* (45–53). Trägårdh’s organ (Tg) distinct, elongate conical.

*Epimeral and lateral podosomal regions*. Apodemes 1, 2, sejugal and 3 well visible. Seven pairs of setiform, smooth epimeral setae observed; setal formula: 1–1–3–2. Setae *3b*, *3c* and *4c* longer than *1a*, *2a*, *4a* and *4b* (Table 1). Discidia (*dis*) triangular. Circumpedal carinae (*cp*) distinct.

*Anogenital region*. Six pairs of genital (*g*_1_–*g*_6_), one pair of aggenital (*ag*), two pairs of anal (*an*_1_, *an*_2_) and three pairs of adanal (*ad*_1_–*ad*_3_) setae setiform, thin, smooth (Table 1). Anterior edge of genital plates with two setae. Adanal setae *ad*_3_ inserted laterally or slightly postero-laterally to lyrifissures *iad*. Postanal porose area (*Ap*, 73–110 × 12–24) transversly oriented, oblong. Ovipositor of typical form for Galumnidae (Ermilov 2010): elongate, narrow (327–369 × 65–69); length of lobes 151–164, length of cylindrical distal part 176–205. Each lobes with four thin, smooth setae: ψ_1_ ≈ τ_1_ (82–98) longer than ψ_2_ ≈ τ_a_ ≈ τ_b_ ≈τ_c_ (36–41). Coronal setae *k* short, thorn-like (12–16).

*Legs*. Morphology of leg segments, setae and solenidia typical for Galumnidae (Ermilov and Anichkin 2011a, 2011b). Formulae of leg setation and solenidia: I (1–4–3–4–20) [1–2–2], II (1–4–3–4–15) [1–1–2], III (1–2–1–3–15) [1–1–0], IV (1–2–2–3–12) [0–1–0]; homology of setae and solenidia indicated in Table 2.

**Table 2.** Leg setation and solenidia of adult *Galumna obvia*

Leg	Trochanter	Femur	Genu	Tibia	Tarsus
I	*v*’	*d*, (*l*), *bv*’’	(*l*), *v*’, σ	(*l*), (*v*), φ_1_, φ_2_	(*ft*), (*tc*), (*it*), (*p*), (*u*), (*a*), *s*, (*pv*), *v*’, (*pl*), *l*’’, *e*, ω_1_, ω_2_
II	*v*’	*d*, (*l*), *bv*’’	(*l*), *v*’, σ	(*l*), (*v)*, φ	(*ft*), (*tc*), (*it*), (*p*), (*u*), (*a*), *s*, (*pv*), ω_1_, ω_2_
III	*v*’	*d*, *ev*’	*l*’, σ	*l*’, (*v*), φ	*(ft*), (*tc*), (*it*), (*p*), (*u*), (*a*), *s*, (*pv*)
IV	*v*’	*d*, *ev*’	*d*, *l*’	*l*’, (*v*), φ	*ft*’’, (*tc*), (*p*), (*u*), (*a*), *s*, (*pv*)

Roman letters refer to normal setae (*e* to famulus), Greek letters to solenidia. Single prime (’) marks setae on anterior and double prime (’’) setae on posterior side of the given leg segment. Parentheses refer to a pseudosymmetrical setae

## Larva, proto- and deutonymph

(Figs 21–30)

**Figures 21–24.**
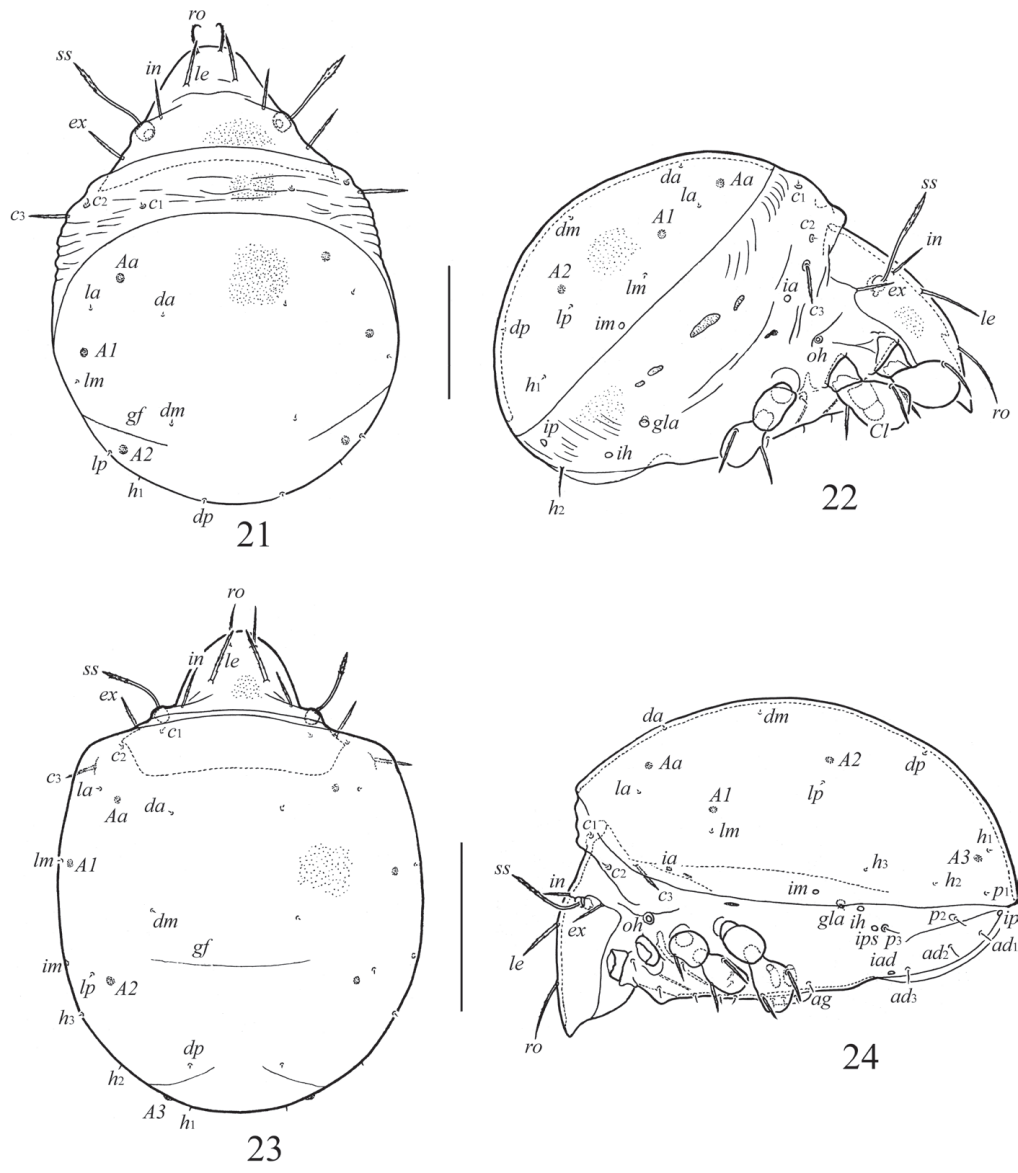
*Galumna obvia*, juvenile instars: **21** larva, dorsal view **22** larva, lateral view (gnathosoma and legs except basal parts not shown) **23** deutonymph, dorsal view **24** deutonymph, lateral view (gnathosoma and and legs except basal parts not shown). Scale bars 100 μm (**21, 22**), 200 μm (**23, 24**).

**Figures 25–33.**
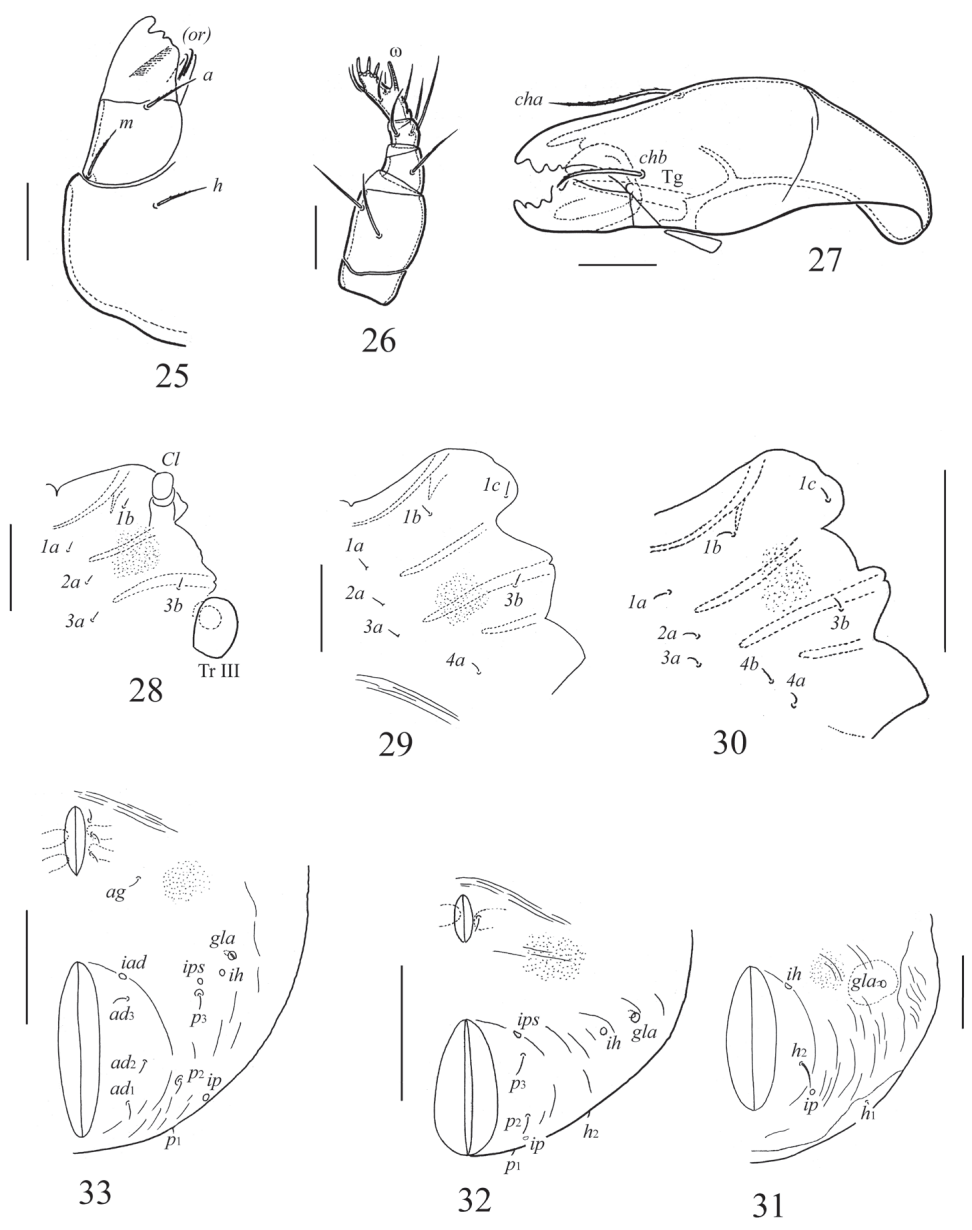
*Galumna obvia*, juvenile instars: **25** subcapitulum, right half, ventral view **26** palp **27** chelicera **28** epimeral region of larva **29** epimeral region of protonymph **30** epimeral region of deutonymph **31** anogenital region of larva **32** anogenital region of protonymph **33** anogenital region of deutonymph. Scale bars 20 μm (**25–27**), 50 μm (**28, 29, 31**), 100 μm (**30, 32, 33**).

*Dimensions*. Length: larva 344–352 (five specimens), protonymph 431, 435 (two specimens), deutonymph 564 (one specimen). Width: larva 246–254, protonymph 332, 336, deutonymph 431.

*Integument*. Prodorsum, gastronotic shield, gnathosoma and legs light brownish; dorsosejugal and epimeral regions and lateral sides colorless to yellowish. Sclerotized body cuticle microfoveolate (diameter foveolae up to 1); soft dorsosejugal, lateral and anogenital regions region with some folds.

*Prodorsum*. Relatively short, about 1/2 length of gastronotic region. Rostrum broadly rounded. Rostral, lamellar, interlamellar and exobothridial setae setiform, barbed, inserted on small tubercles. Sensilli with long stalk and weakly developed, lanceolate, barbed head. Relative length of prodorsal setae: *ss* > *ro* > *le* > *in* ≈ *ex*; measurements compared in Table 1.

*Gastronotic region*. Dorsal gastronotic region with large, well-bordered shield (macrosclerite) in all juvenile instars. Transversal gastronotic furrow present (*gf*), poorly visible. Lateral sides with several small, elongate sclerites (smaller and weakly visible in nymphs than in larva). Larva with 11 pairs of gastronotic setae, proto- and deutonymphs with 15 pairs. Setae *c*_1_–*c*_3_ and *p*_2_–*p*_3_ (in deutonymph) on small sclerites. Gastronotic shield with seven pairs of setae (*da*, *dm*, *dp*, *la*, *lm*, *lp*, *h*_1_) in larva, 10 pairs (*da*, *dm*, *dp*, *la*, *lm*, *lp*, *h*_1_–*h*_3_, *p*_1_) in proto- and deutonymphs. Gastronotic setae *c*_3_ longest, straight, barbed; *h*_2_ in larva shorter, setiform, slightly barbed; *p*_2_, *p*_3_ in proto- and deutonymph setiform, smooth; other setae very short, thin, smooth (Table 1). Porose areas rounded, poorly visible: larva with three pairs (6–8, *Aa*, *A1*, *A2*), proto- (8) and deutonymph (12) with four pairs (*A3* present additionally). Cupules *ia*, *im* and *ip* clearly visible. Humeral organ (*oh*) well developed.

*Gnathosoma*. Similar to that of adult instar.

*Epimeral region*. Setal formulae for epimeres: larva 3–1–2 (larval seta *1c* scale-like, covering tip of retracted Claparède’s organ); protonymph 3–1–2–1; deutonymph 3–1–2–2. Epimeral setae setiform, smooth (Table 1).

*Anogenital region*. Ontogenetic genital, aggenital, adanal, anal formulae (larva to deutonymph): 0–1–3, 0–0–1, 0–0–3, 0–0–0, respectively. All setae setiform, thin, smooth (Table 1). Paraproctal setae absent. Cupules *ih*, *ips*, *iad* and opisthonotal gland openings clearly visible, appearing in normal ontogenetic pattern.

*Legs*. Ontogeny of leg setae and solenidia given in Table 3.

**Table 3.** Development of leg setation of *Galumna obvia* during ontogeny.

	Trochanter	Femur	Genu	Tibia	Tarsus
Leg I					
Larva	–	*d*, *bv*’’	(*l*), σ	(*l*), *v*’, φ_1_	(*ft*), (*tc*), (*p*), (*u*), (*a*), *s*, (*pv*), (*pl*), *e*, ω_1_
Protonymph	–	–	–	–	ω_2_
Deutonymph	–	(*l*)	–	φ_2_	–
Leg II					
Larva	–	*d*, *bv*’’	(*l*), σ	*l*’, *v*’, φ	(*ft*), (*tc*), (*p*), (*u*), (*a*), *s*, (*pv*), ω_1_
Protonymph	–	–	–	–	–
Deutonymph	–	*l*’’	–	*l*’’	ω_2_
Leg III					
Larva	–	*d*, *ev*’	*l*’, σ	*v*’, φ	(*ft*), (*tc*), (*p*), (*u*), (*a*), *s*, (*pv*)
Protonymph	–	–	–	–	–
Deutonymph	*v*’	–	–	*v*’’	–
Leg IV					
Protonymph	–	–	–	–	*ft*’’, (*p*), (*u*), (*pv*)
Deutonymph	–	*d*, *ev*’	*d*, *l*’	*v*’, φ	(*tc*), (*a*), *s*

See Table 1 for explanations. Setae are listed only for the instar in which they first appear.

## Comparison

The adult specimens of *Galumna obvia* collected in Finland correspond to earlier redescriptions (Willmann 1931; Zachvatkin 1953; Aoki 1966; Wallwork 1977; Mahunka 1992; Pérez-Íñigo 1993; Mahunka and Mahunka-Papp 1995; Weigmann 2006; Bayartogtokh 2011) and material in the personal collection of G. Weigmann in terms of general appearance: reatively large body; short, thin interlamellar setae; long, setiform rostral and lamellar setae; sensilli with weakly developed, lanceolate head; four pairs of notogastral porose areas, *Aa* and *Ap* being transversely elongate; and short ventral setae. However, the adults from Finland are distinguishable by the presence of a long furrow on the ventral plate, which is not mentioned in other descriptions. We consider this difference as intraspecific variability (perhaps geographical), which should be taken into account in any future identification of this species.

Juvenile instars (larva, proto- and deutonymph) of *Galumna obvia* correspond to those of other Galumnidae in many characters (cf. Michael 1884; Grandjean 1928, 1935, 1953, 1956a; Cooreman 1941; Zachvatkin 1953; Sengbusch 1954; Travé 1955, 1970; Wallwork 1965; Woodring 1965; Rockett and Woodring 1966; Seniczak 1972; Grishina 1977, 1982; Bernini 1984; Seniczak and Seniczak 2007; Seniczak et al. 2012). These include: gastronotum covered by gastronotic shield (macrosclerite); gastronotic setae not on shield inserted on microsclerites; prodorsal setae long or medium size; sensilli long, lanceolate; larva with 11 or 12 pairs (seven pairs inserted on gastronotic shield) and nymphal instars – with 15 pairs (10 pairs inserted on gastronotic shield) of gastronotic setae; gastronotic shield with three (in larva) or four (in nymphal instars) pairs of weakly visible porose areas); humeral organ present; genital formula 0–1–3–5, aggenital formula 0–0–1–1, adanal formula 0–0–3–3, anal formula 0–0–0–2. Juvenile instars of *Galumna obvia* can be distinguished from those of other Galumnidae as follows.

- From *Pilogalumna* (*Pilogalumna crassiclava*, *Pilogalumna ornatula*, *Pilogalumna tenuiclava*) by: the length of prodorsal setae (*ro* longest in *Galumna obvia* versus *in* longest in *Pilogalumna* species); length of gastronotic setae of *c*-series (*c*_3_ of medium size, *c*_1_ and *c*_2_ short, *c*_3_ > *c*_1_ ≈ *c*_2_ in *Galumna obvia* versus *c*_3_ and *c*_2_ of medium size, *c*_1_ short, *c*_3_ > *c*_2_ > *c*_1_ in *Pilogalumna* species); and number of gastronotic setae and length of setae *h*_1_ in larval instar (11 pairs – *h*_3_ absent, *h*_1_ short in *Galumna obvia* versus 12 pairs – *h*_3_ present, *h*_1_ of medium size in *Pilogalumna* species).
- From *Acrogalumna* (*Acrogalumna longipluma*) by: the length of prodorsal setae (*ro* longest in *Galumna obvia* versus *le* longest in *Acrogalumna longipluma*); the length of gastronotic setae of *c*-serie (*c*_3_ of medium size, *c*_1_ and *c*_2_ short, *c*_3_ > *c*_1_ ≈ *c*_2_ in *Galumna obvia* versus *c*_3_ and *c*_2_ of medium size, *c*_1_ short, *c*_3_ > *c*_2_ > *c*_1_ in *Acrogalumna longipluma*).
- From *Allogalumna* (*Allogalumna alamellae*) by: the length of prodorsal setae (*ro* > *le* > *in* in *Galumna obvia* versus *in* > *ro* ≈(>) *le* in *Allogalumna alamellae*); the length of gastronotic setae of *c*-serie (*c*_3_ of medium size, *c*_1_ and *c*_2_ short, *c*_3_ > *c*_1_ ≈ *c*_2_ in *Galumna obvia* versus *c*_3_ and *c*_2_ of medium size, *c*_1_ short, *c*_3_ ≈ *c*_2_ > *c*_1_ in *Allogalumna alamellae*).
- From *Galumna* species: from *Galumna alata* by the length of prodorsal setae (*ro* > *le* > *in* in *Galumna obvia* versus *in* > *ro* > *le* in larva, *in* ≈(>) *le* > *ro* in nymphal instars in *Galumna alata*), the length of gastronotic setae of *c*-series (*c*_3_ of medium size, *c*_1_ and *c*_2_ short, *c*_3_ > *c*_1_ ≈ *c*_2_ in *Galumna obvia* versus *c*_3_ and *c*_2_ of medium size, *c*_1_ short, *c*_2_ > *c*_3_ > *c*_1_ in *Galumna alata*); from *Galumna zachvatkini* by the length of gastronotic setae (*c*_3_ of medium size, *c*_1_, *c*_2_ and other dorsal setae short, *c*_3_ > *c*_1_ ≈ *c*_2_ in *Galumna obvia* versus *c*_1_, *c*_2_, *c*_3_ and other dorsal setae well developed, of medium size, *c*_1_ ≈ *c*_2_ ≈*c*_3_ in *Galumna zachvatkini*), and number of gastronotic setae in larval instar (11 pairs – *h*_3_ absent in *Galumna obvia* versus 12 pairs – *h*_3_ present in *Galumna zachvatkini*).
- From *Pergalumna* (*Pergalumna nervosa*) by: the length of prodorsal setae (*ro* > *le* > *in* in *Galumna obvia* versus *le* ≈(>) *ro* > *in* *Pergalumna nervosa*); the length of gastronotic setae of *c*-serie and number of gastronotic setae and length of setae *h*_1_ in larval instar (*c*_3_ of medium size, *c*_1_ and *c*_2_ short, *c*_3_ > *c*_1_ ≈ *c*_2_, 11 pairs setae present – *h*_3_ absent in *Galumna obvia* versus *c*_3_ and *c*_2_ of medium size, *c*_1_ short, *c*_3_ ≈ *c*_2_ > *c*_1_,12 pairs setae present – *h*_3_ present in *Pergalumna nervosa*).

Thus, the diagnostic morphological characters of Galumnidae juvenile instars are not numerous and can be summarized as: the length of rostral, lamellar and interlamellar setae; the number of gastronotic setae in larval instar; the length of gastronotic setae of *c*-series, *dp*, *h*_1_; the presence or absence of a transverse furrow on gastronotic shield and genital and adanal macrosclerites on the ventral side in nymphal instars); and body size.

## Taxonomic discussion: the position of seta *le* in species of *Galumna* and *Pergalumna*

In the Finnish population of *Galumna obvia*, the lamellar seta (*le*) inserts medial to the lamellar line, at a distance of about 5 µm; no distinct variability is observed. The conventional definition of the genus *Galumna* includes the differential character “lamellar seta on (at) the lamellar line” in contrast to the definition of the genus *Pergalumna* Grandjean, 1936, originally as subgenus with the differential character “lamellar seta in some distance medially to the lamellar line” (Grandjean 1936; cf. keys of Sellnick 1960, Pérez-Iñigo 1993, Weigmann 2006). Following a strict interpretation of the *le* position, the Finnish population of *Galumna obvia* could be regarded as a *Pergalumna* species. To resolve this will require a detailed reevaluation of *Galumna* and a comparison with *Pergalumna*.

We compared the characters of the Finnish population of *Galumna obvia* with those in some other European populations, especially from northwest to northeast of Germany; and we found no convincing character combinations to exclude the Finnish population from *Galumna obvia*, regarding body size (indicated for German populations with 705–845 µm total body length: Weigmann 2006), setation, shapes and positions of porose areas on notogaster, sensillus shape and other characters which are used in literature to define *Galumna obvia*. Concerning the position of setae *le* relative to the lamellar line, we found remarkable variability. In some populations or specimens, the seta *le* has some distance, continuously up to 6 µm, in median direction from the lamellar line; in other populations or single specimen *le* inserts strait at the lamellar line on the median side (cf. Figs 37 and 38): In populations “Oder valley” (n=2) – 0–1 µm, “Berlin 1” (n=3) – 5–6 µm, “Berlin 2” (n=6) – 1–5 µm, “Berlin 3” (n=3) – 0–1 µm, “Oldesloe” (n=6) – 0–4 µm. In all populations we observed a small variability, partly with different ranges. Unfortunately, we have no information about Berlese’s typical population in Italy.

**Figures 34–36.**
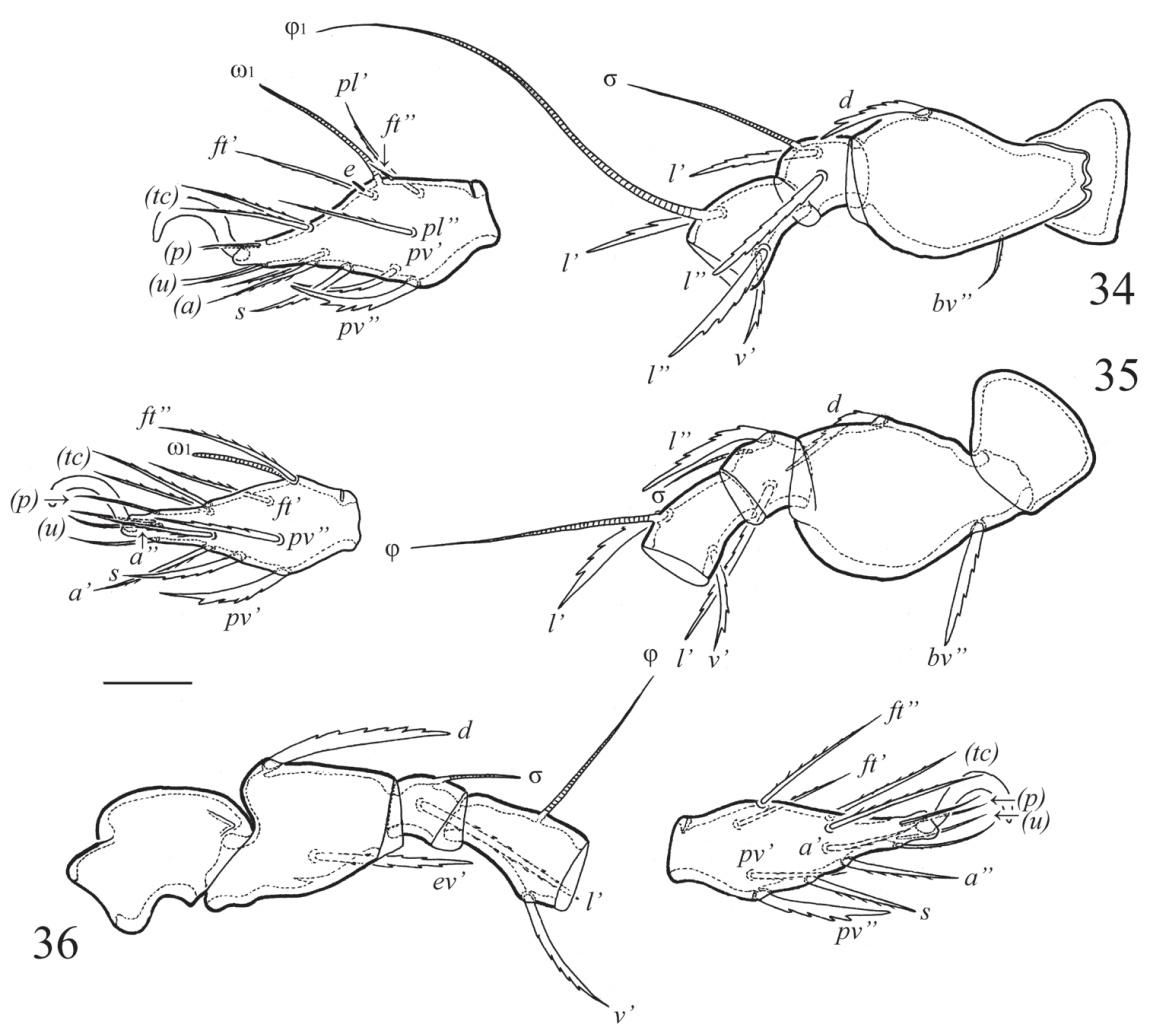
*Galumna obvia*, juvenile instars: **34** leg I, left, antiaxial view **35** leg II, left, antiaxial view **36** leg III, right, paraxial view. Scale bar 20 μm.

**Figures 37–41.**
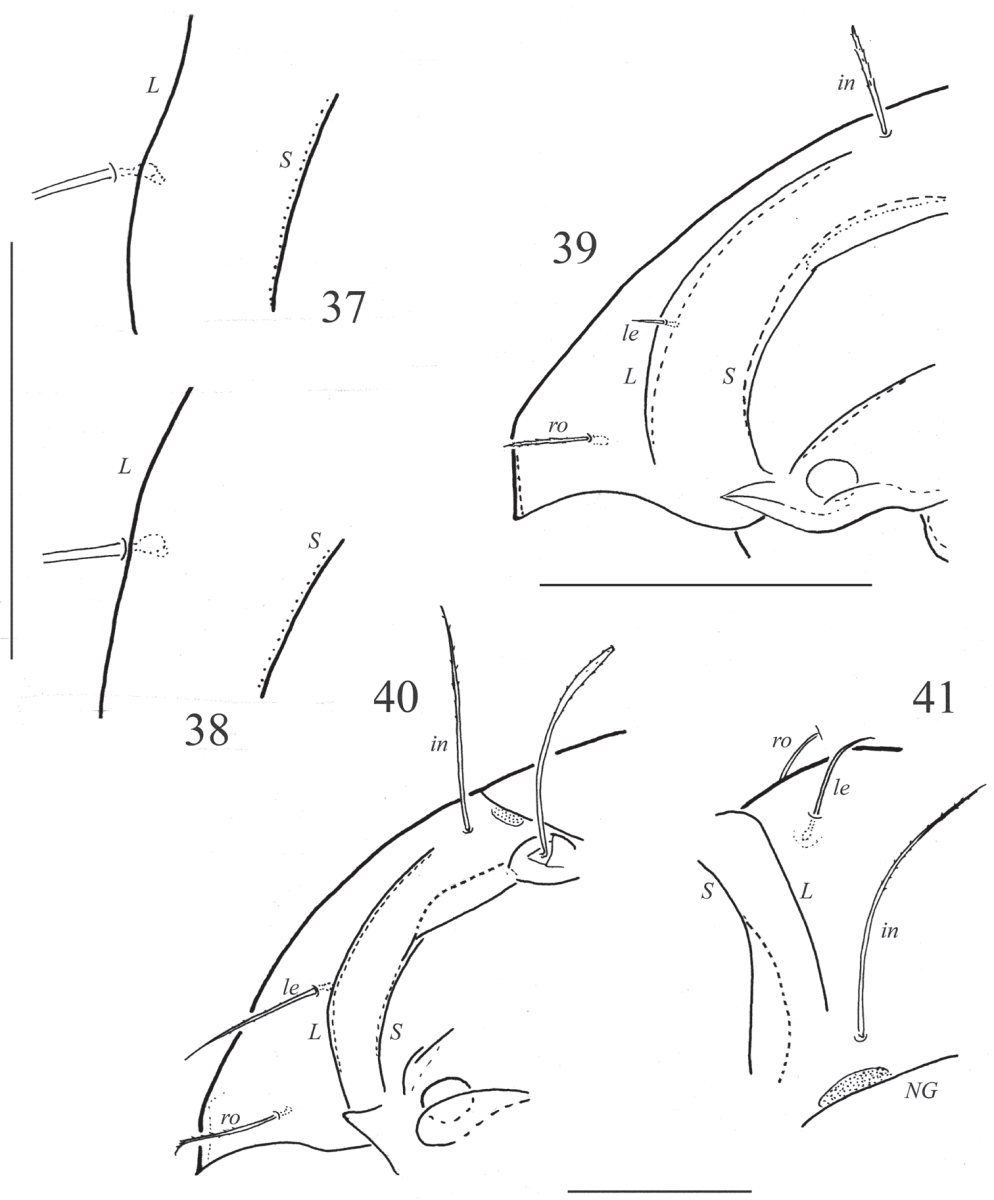
Adults of Galumnidae: **37** *Galumna obvia*, lamellar region of prodorsum, lateral view (specimen from Berlin) **38** *Galumna obvia*, lamellar region of prodorsum, lateral view (specimen from Oder Valley, North-East Germany) **39** *Galumna paragibbula*, lateral view of prodorsum **40** *Pergalumna nervosa*, lateral view of prodorsum **41** *Pergalumna nervosa*, dorso-frontal view of left part of prodorsum (depressed mounted specimen). Abbreviation: *NG*– notogastral shield. Scale bar 100 μm.

These slight differences of the position of seta *le* raise a similar question with regard to other *Galumna* species. Since species descriptions, redescriptions and illustrations are often not sufficiently precise, we give only selective examples. In the type species of *Galumna*, *Galumna alata* (Hermann, 1804), Grandjean (1936: 97) figured in his very accurate redescription the setae *le* on the lamellar line. In material of one of the coauthors (G.W.; location “Berlin 3”) of *Galumna alata*, the insertion of *le* is a short distance laterally from the lamellar line. The latter position of *le* is described in several other *Galumna* species, e.g. *Galumna asiatica* Grishina, 1981 (Bayartogtokh and Weigmann 2005); *Galumna dimorpha* Krivolutsky, 1952 (Bayartogtokh and Weigmann 2005); *Galumna gibbula* Grandjean, 1956 (Grandjean 1956b: p. 144, 145 as *Galumna tarsipennata gibbula*); *Galumna lanceata* Oudemans, 1900 (from three urban sites “Berlin 4”, actually studied within this project); *Galumna paragibbula* Weigmann 2011 (cf. Fig. 39); *Galumna tarsipennata* Oudemans, 1914 (Travé 1970 from France; actually studied (G.W.) from floodplain forest, South Portugal).

Comparing the position of seta *le* in strict lateral aspect in *Pergalumna nervosa* (Fig. 40) and in *Galumna obvia* (Figs 6, 37), there seems to be less difference: in both species the seta seems to be inserted a short distance medially from the lamellar line. Yet in dorso-frontal aspect without parallactic error, the distance between *le* and the lamellar line is about 27 µm in *Pergalumna nervosa* (Fig. 41), in *Galumna obvia* at most 6 µm.

As a preliminary conclusion, most studied *Galumna* species have the seta *le* inserted a short distance lateral to the lamellar line; in *Galumna alata* the seta is positioned on the line or lateral to it. *Galumna obvia* is the only species observed with a *le* insertion medial to the lamellar line or in some specimens on it. The latter two species both show some variability of the *le* insertion.

The differentiation of the genera *Galumna* and *Pergalumna*, defined by Grandjean (1936) by means of the insertion of seta *le*, is called into question by the variable character state in *Galumna obvia*. This single differentiation character is of questionable value to discriminate genera as monophyletic entities, and the character is a simple one with a tendency to variability. Nevertheless, it is convenient to split the *Galumna*-*Pergalumna* complex in two parts for determination in keys: in *Galumna* “*le* is inserted on the lamellar line”, in *Pergalumna* “medially at an obvious distance”. We propose to maintain both genera provisionally until a desirable multifactorial phylogenetic analysis is performed.

An analogous case in the family Malaconothridae relates to the single argument to differentiate *Malaconothrus* Berlese, 1904 from *Trimalaconothrus* Berlese, 1916, by the typological characters “monodactylous or tridactylous legs”. This character state is easy to distinguish but obviously without phylogenetical value. Colloff and Cameron (2013) provided a multifactorial analysis for several species of both genera. They found no reasonable pattern to confirm either genera as monophyletic: as a result *Trimalaconothrus* was considered a junior synonym; *Malaconothrus* and *Tyrphonothrus* Knülle, 1957, could be established as valid taxa without the number of claws being a key character.
